# QTL and Candidate Genes: Techniques and Advancement in Abiotic Stress Resistance Breeding of Major Cereals

**DOI:** 10.3390/ijms24010006

**Published:** 2022-12-20

**Authors:** Sujitra Raj Genga Raj, Kalaivani Nadarajah

**Affiliations:** Department of Biological Sciences and Biotechnology, Faculty of Science and Technology, Universiti Kebangsaan Malaysia, Bangi 43600, Selangor, Malaysia

**Keywords:** Quantitative trait locus/loci (QTL), abiotic stresses, breeding programs, cereals, rice, wheat, maize, candidate genes, modern and conventional breeding techniques

## Abstract

At least 75% of the world’s grain production comes from the three most important cereal crops: rice (*Oryza sativa*), wheat (*Triticum aestivum*), and maize (*Zea mays*). However, abiotic stressors such as heavy metal toxicity, salinity, low temperatures, and drought are all significant hazards to the growth and development of these grains. Quantitative trait locus (QTL) discovery and mapping have enhanced agricultural production and output by enabling plant breeders to better comprehend abiotic stress tolerance processes in cereals. Molecular markers and stable QTL are important for molecular breeding and candidate gene discovery, which may be utilized in transgenic or molecular introgression. Researchers can now study synteny between rice, maize, and wheat to gain a better understanding of the relationships between the QTL or genes that are important for a particular stress adaptation and phenotypic improvement in these cereals from analyzing reports on QTL and candidate genes. An overview of constitutive QTL, adaptive QTL, and significant stable multi-environment and multi-trait QTL is provided in this article as a solid framework for use and knowledge in genetic enhancement. Several QTL, such as DRO1 and Saltol, and other significant success cases are discussed in this review. We have highlighted techniques and advancements for abiotic stress tolerance breeding programs in cereals, the challenges encountered in introgressing beneficial QTL using traditional breeding techniques such as mutation breeding and marker-assisted selection (MAS), and the in roads made by new breeding methods such as genome-wide association studies (GWASs), the clustered regularly interspaced short palindromic repeat (CRISPR)/Cas9 system, and meta-QTL (MQTL) analysis. A combination of these conventional and modern breeding approaches can be used to apply the QTL and candidate gene information in genetic improvement of cereals against abiotic stresses.

## 1. Introduction

Abiotic stresses are major threats to grain cereals. Cereals are increasingly subjected to a range of abiotic stress combinations as a consequence of global warming and climate change, which have a significant influence on their growth and yield [1]. Global climate change leads to severe abiotic stress on crops due to the continued rise in air temperature and atmospheric CO_2_ levels, which alters rainfall patterns and distribution [2]. As a result, abiotic stresses are expected to diminish total crop production by more than 50% and have a negative influence on 70% of main food crop yields [3]. Drought, extreme cold, salinity, submergence, and metal toxicity are among the major stresses faced by cereals [4,5].

Rice (*Oryza sativa* L.), the world’s most consumed grain, is extremely sensitive to these major abiotic stressors [6]. Similarly, these abiotic stressors have significant effects on maize and wheat’s growth and development by drastically changing nutritional contents, lowering grain quality, and reducing production [7,8,9]. While growth recovery in cereals is possible when the stress is short term, low intensity, and involves stress-resilient cereals, prolonged exposure, on the other hand, may result in drastic changes in metabolic activities and phenological stages that could severely hamper yield [1]. For instance, extreme temperatures affect plant growth, maturation, and geographical distribution, along with crop quality and yield. Drought stress in the rhizosphere impedes plant development, inhibits nutrient absorption, and results in significant losses of grain yield [2]. Cold stress, on the other hand, can trigger phenotypic changes in cereals, such as decreased leaf growth, withering, and chlorosis. This can lead to necrosis, and eventually cause cellular collapse or even death of the entire plant [10]. Salinity stress can increase the generation of reactive oxygen species (ROS) which harm cellular components through oxidation of proteins, lipids, and nucleic acids, as well as induce cell death [11]. Further, high levels of metal in soil have reduced soil fertility and agricultural production by affecting the soil physicochemistry and the microbial activity in the soil [12]. As a consequence of climate change, major abiotic stresses affect agricultural productivity and food stability [13].

Quantitative trait locus (QTL) mapping has emerged as a useful plant breeding approach to unravel complex trait architecture and identify candidate genes [14]. As a result, the significant and consistent QTL revealed in diverse genetic and environmental backgrounds may be valuable for future gene cloning, and detection of breeding-relevant molecular markers. Traditional plant breeding methods such as mutation breeding and marker-assisted selection (MAS) using molecular markers have expanded the genetic resources available for cereal development through introgression of desirable characteristics [15]. New approaches such as genome-wide association studies (GWASs), clustered regularly interspaced short palindromic repeats (CRISPR), next-generation sequencing (NGS), and meta-analysis will undoubtedly improve the efficiency and effectiveness of plant breeding.

Due to the accelerating global climate change, this review serves as an important source of information, highlighting beneficial QTL and candidate genes involved in resistance against primary abiotic stresses in these cereals, while identifying techniques, progress, and the issues faced in selection and introgression of the QTL and candidate genes. This review focuses on some important and stable QTL related to important abiotic stresses in cereals, while providing methods for identification of QTL and genes relevant to enhancing agronomic traits in these three key cereal crops. Identifying multiple genes and QTL improves many agriculturally significant properties such as yield, quality, growth, and stress management. Most published manuscripts on breeding have been focused on a single cereal, provided a general overview of plant breeding, or looked into breeding related to one specific stress or influence. In contrast, here, we have focused and combined the information for the three main cereals of the world and collated data for the main five abiotic stresses faced by these main cereal crops.

This review can be a comprehensive guide on developing high-yielding cereal varieties with abiotic stress tolerance that could considerably help control yield losses. Plant scientists and breeders working on cereal improvement against climate and environmental changes may find it useful for yield improvement.

## 2. Major Abiotic Stresses Affecting Cereals

### 2.1. Drought and Heat Stress

Drought stress is a significant abiotic stress that results in substantial agricultural losses. The frequency and intensity of drought induced by climate change will continue to be of global concern [16]. Temperatures beyond the threshold range (28 °C to 32 °C) and an increase of roughly 3–4 °C can limit a crop’s capacity to adapt, and result in reduced yield of up to 35% [17]. In cereals, the combination of both water scarcity and heat stress leads to significant impairment of stomatal conductance, water relations, CO_2_ uptake, and photosynthetic pigments. This results in the disruption of reproduction or seed maturation processes; hence contributing to decreased cereal yield [18]. Hence, a variety of additional drought-related traits, such as photosynthesis [19], cereal body architecture [20], osmotic characteristics [21], and resource allocation to roots or shoots [22,23,24,25] have also been explored to better understand drought resistance. Since the health and activity of the roots have a direct influence on the cereals’ capacity to collect water, this organ remains the foremost critical components for enhancing capacity under drought stress [26].

### 2.2. Extreme Cold Stress

Cereals are also exposed to extreme cold stress. The physiology of grains is also influenced by cold stress. Cold damage is common in northern China as well as many other cereal-growing regions across the world during the winter, leading to millions of tons of losses each year [27,28]. Cold stress, especially freezing and chilling temperatures, is among major abiotic stresses for global cereal productivity. Due to low soil temperatures, roots cannot absorb water. As the water permeability decreases, the cereal water status, stomatal conductance, photosynthesis, growth, and, lastly, productivity are impacted negatively [16]. It is also observed that early in the vegetative cycle, cold stress in cereals inhibits growth and increases seedling mortality. This reduces growth, delays heading, and induces leaf wilting, yellowing, or staining, which in turn reduce yield [29].

### 2.3. Flooding Stress

There are two types of flooding occurrences. The first is waterlogging, which affects just the root system within the soil; the second is submergence, which involves the submergence of the entire plant in water. The oxygen content quickly drops in flooded cereal without continuous photosynthesis, resulting in hypoxia [13]. The growth of cereal is restricted by the lack of oxygen in the soil, which lowers agricultural output [30]. Submergence stress is more dangerous than waterlogging because excessive water at any development stage in submergence stress can cause crop harm and yield loss compared to waterlogging that mainly affects the root development stage. The early effect of submergence stress is stunted seedling growth, lower germination percentages, and poor crop establishment [31]. On the other hand, waterlogging increases the anaerobic respiration of the cereals, raises their energy use, and ultimately limits their growth [30].

### 2.4. Salinity Stress

Salinity is a significant abiotic stress that has a detrimental effect on the development and yield production of cereals worldwide [32] by causing numerous physiological and biochemical changes in cereals, including osmotic stress, ionic imbalances, and secondary stress [33]. Many important components of cereals are affected by salinity stress, including physiological, morphological, ultrastructural alterations in cells, biochemical production, and the activation of numerous other molecular activities [34]. The early stage of salinity stress is known as hyperosmotic stress. In this stage, the ability of root systems to absorb water declines and water loss from leaves accelerates [35]. Salinity stress in the soil reduces the water potential between the soil and the leaves. Osmotic stress eventually results in the disruption of plant water relationships and reduces plant turgor. Salinity causes an ionic imbalance by causing large amounts of ions (Na^+^ and Cl^−^) to accumulate and prevent the uptake of K^+^ and Ca^2+^ [36]. Salinity stress has resulted in the activation of complicated adaptive responses that aim to synchronize the ions to reduce hyperosmolarity and restore cellular ionic homeostasis [37]. According to researchers, salinity has been shown to affect total cereal development by altering intricate interactions in nutrient absorption and accumulation, hormonal imbalance, and oxidative stress [38,39,40].

### 2.5. Heavy Metal Stress

Heavy metal toxicity is a result of agricultural pollutants that affects crops in many regions of the world. This pollution may be caused by long-term usage of phosphate fertilizers, industrial waste, sewage sludge application, and improper irrigation practices [41]. As a consequence of agricultural practices worldwide, heavy metal poisoning has become a global concern to all humans. The build-up of heavy metals wreaks havoc on agricultural land fertility [42]. Heavy metals are thought to induce oxidative damage at the cellular level by acting as important metal ions exchangers or by inhibiting functional groups. Redox active metals such as iron (Fe) and copper (Cu) form ROS directly through redox processes, while other metals such as lead (Pb), cadmium (Cd), nickel (Ni), aluminum (Al), manganese (Mn), and zinc (Zn) generate ROS indirectly [43].

While a substantial amount of research into the physiology and genetics of cereal responses to abiotic stress has been conducted, questions still remain on how these stresses affect the physiology and biology of these crops. Therefore, there is necessity to further study the complexities of cereal response to abiotic stress. The following section will discuss the numerous studies related to major QTL and candidate genes in rice, maize, and wheat with reference to the above abiotic stress tolerance.

## 3. Current Advances in Abiotic Stress Breeding of Cereals: QTL and Candidate Genes

Since abiotic stresses affect the growth and development of cereals, it is not surprising to find hundreds of studies and reviews of QTL analysis that report on the detrimental impacts of these stressors. Identification of QTL that control traits for abiotic stress involves a sequence of steps. Firstly, (i) mapping populations in which the traits of interest linked with the specific abiotic stress are identified, (ii) identification of polymorphic markers, (iii) genotyping of mapping populations using polymorphic markers, (iv) precise phenotyping based on the abiotic stress tolerance-correlated traits, and finally (v) QTL mapping based on genotypic and phenotypic data [44]. Full-sib F1, F2, doubled-haploid lines (DHLs), backcross 1 (BC1), recombinant inbred lines (RILs), and near-isogenic lines (NILs) are the most common segregating populations used for QTL mapping [45].

Molecular breeding approaches, especially by targeting candidate genes for genetic engineering and the production of transgenic lines, can be used to exploit QTL for crop improvement. The discovery of candidate genes is crucial for our understanding of the molecular and physiological mechanisms behind drought tolerance in cereals. This information may be used not only in transgenic studies but may be utilized in development of markers for selection and screening purposes [46]. Further, the function assigned to genes may also be utilized in marker-assisted selection (MAS) to improve varieties and speed up plant breeding under abiotic stress by manipulating either a single gene or a pyramid of favorable QTL alleles [47]. In addition, synteny among cereals opens up a pathway to understand the genome-wide relationships among the QTL or genes that are relevant to the particular stress adaptation and trait improvement in crops [48].

### 3.1. Major Abiotic Stress-Related QTL and Candidate Genes in Rice

Rice is widely grown throughout Asia, which accounts for over 90% of global agriculture. It is grown in a variety of ecologies, such as highlands, lowlands, and deep waters [47]. Rice is the staple food for half of the world’s population, contributing 30–80% of daily calories to mainly the Asian population [22]. Abiotic stressors such as drought, salinity, submergence, severe temperatures, and heavy metal toxicity challenge and affect rice yield [22]. As rice is a major cereal crop and food source, extensive research has been carried out on the QTL and candidate genes for abiotic stress resistance in rice. In our opinion, focusing on the QTL of traits related to yield such as grain weight and grain number per panicle is an efficient strategy for rice breeding against abiotic stress.

#### 3.1.1. Drought and Heat Stress

Drought-tolerant rice varieties IR64, Sabitri, Swarna, Mahsuri, and Sambha have been cultivated for testing and distribution in state and national trials in many countries [49]. In addition, many traditionally cultivated landraces of rice, including Khao Dawk Mali, Azucena, Dular, Rayada, Bala, Apo, Nam Sagui 19, Nagina 22, Aday Sel, Dehula, Moro berekan, Huma Wangi Lenggong, Siam Pilihan, Chianung Sen Yu, Kashmir Basmati, and MR142 have been reported to be highly drought tolerant. Researchers may examine these drought-tolerant varieties to identify underlying genetic potential and QTL for use in breeding programs. The introduction of drought yield QTL using marker-assisted breeding (MAB) could speed the development of drought-tolerant rice varieties. This approach has been successfully used by Shamsudin et al. (2016) in pyramiding three drought yield QTL, qDTY 2.2, qDTY 3.1, and qDTY 12.1, by MAB into the Malaysian high-quality rice cultivar MRQ74 [50]. Zhao et al. (2016) used a cross between heat-susceptible Sasanishiki (*japonica*) and heat-tolerant Habataki (*indica*) rice varieties derived from chromosome segment substitution lines to map 11 QTL on chromosomes 1, 2, 3, 4, 5, 7, 8, 10, and 11 using simple sequence repeat (SSR) markers for flowering time, fertility of spikelet, and shedding of pollen under heat stress, resulting in the discovery of three QTL, qPSLht4.1, qPSLht7, and qPSLht10.2. Among these three QTL, qPSLht4.1 confers heat tolerance under a variety of temperature regimes, and thus has the potential to be successfully employed for improved pollen shedding and pollen growth on stigma [20]. Based on the assessment of RILs produced from Cocodrie and N-22, eight QTL grain yield traits were discovered under drought. The majority of these QTL were found on chromosome 1, suggesting that it might be a potential carrier of drought stress tolerance [51]. Selamat and Nadarajah (2021) reported that chromosomes 1, 2, and 3 in rice featured a large number of QTL and many rice traits that respond to drought tolerance and resistance. A few significant proteins related to drought stress were identified in this study, which are abscisic acid-insensitive protein 5 (ABI5), the G-box binding factor 4 (GBF4), protein kinase pinoid (PID), histidine kinase 2 (AHK2), protein related to autophagy 18A (ATG18A), mitochondrial transcription termination factor (MTERF), aquaporin PIP 1-2, protein detoxification 48 (DTX48), and inositol-tetrakisphosphate 1-kinase 2 (ITPK2) [52]. Vikram et al. (2011) discovered a major and stable QTL, qDTY1.1, for the grain yield characteristic on rice chromosome 1. This QTL is present in all three F3:4 mapping populations that were created by crossing the drought-tolerant cultivar N22 with the popular mega-varieties Swarna, IR64, and MTU1010. It is identified that this is the first major QTL that appears consistently across several genetic backgrounds that affects grain yield under both reproductive stress and non-stress conditions [53]. In another study, a QTL called qDTY12.1 on chromosome 12 with a consistent and stable impact in two environments in the Philippines and Nepal was shown to be strongly linked with grain yield under reproductive-stage drought stress [54]. This particular QTL on chromosome 12 has also been reported in an earlier study. A hybrid between cultivars Vandana and Way Rarem resulted in the detection of the major QTL qtl12.1 on chromosome 12 for grain yield under stress [55]. This major QTL has been used in an introgression study recently. New drought-tolerant MRQ74 and MR219 pyramided lines were evaluated in a study by Mohd Ikmal et al. (2019) to determine the impact of various qDTY combinations on morphological and agronomical characteristics under drought stress and non-stress conditions. Higher grain yield was discovered in pyramided qDTY12.1 when morphological and agronomical features were improved, either alone or in combination with other qDTYs. Due to its consistent effect on morphological, agronomical, and grain yield traits across populations under both drought stress and non-stressed environments, qDTY12.1 is considered one of the most significant drought qDTYs [56].

*DRO1* is a major QTL that controls rice deep rooting and has been cloned. The duplicated and validated drought-adaptive gene *DRO1* in rice, which is known to affect the angle of root development under drought stress, is also associated with high yields A recent study revealed that the *DRO1*-introgressed lines had higher photosynthetic rates and grain filling, resulting in better yield in these lines. Drought resistance in rice can thus be obtained by transferring backcross-mediated *DRO1* into shallow-rooting rice cultivars [57]. According to Siddiqui et al. (2021), this gene is located on rice chromosome r9 (chromosome 9), which has a syntenic relationship with chromosome w5 (chromosome 5) in wheat, chromosome m10 (chromosome 10) in maize, chromosomes b5 (chromosome 5H) and b7 (chromosome 7H) in barley, and chromosome s2 (chromosome 2) in sorghum. All of these syntenic chromosomal locations have been connected to root-related drought stress adaptation. DRO1 homologs may occur in other economically important cereal crops, such as wheat, barley, maize, and sorghum, and comparative genomics may be exploited to increase root-related drought stress responses in cereals [48]. A comparable gene that encodes the NAC protein was revealed as a potential miRNA target in a prior work on rice, and its down-regulation suggests a role in drought tolerance [58]. Drought tolerance in rice results in down-regulation of genes that encode the NAC A/B superfamily protein. Overexpression of the drought-inducible AP2/ERF family TF gene, *OsERF48*, results in an overextended and denser root structure in transgenic cereals [59]. In order to increase the ability of indica rice to withstand drought, researchers have looked at the co-expression of DREB2A or APX and found that co-expression of DREB2A and APX can improve drought tolerance in rice plants to mitigate the effects of climate change [60].

#### 3.1.2. Cold Stress

More than 100 QTL for cold tolerance were identified by Liang et al. (2018), notably in the booting stage. However, only few of them were verified to be stable QTL across settings, despite further advancement in the molecular genetic analysis of low-temperature stress tolerance in rice [61]. By pyramiding a cold tolerance QTL that promotes cold stress adaptation during the pre-reproductive period, a cold-tolerant rice line in a Bhutanese rice variety, ‘Kuchum’, was produced. In the introgressed area of rice chromosome 4 in Norin-PL8 in NILs, the QTL was fine mapped, revealing qCT-4 associated with cold resistance. This suggests that causal QTL introgression is an efficient way to improve a particular quantitative feature [62]. Using F2 and BC1F_2_ populations from crosses between Ukei 840 and Hitomebore, Shirasawa et al. (2012) discovered a QTL for cold tolerance on chromosome 3 of rice. The qLTB3 region identified for cold tolerance in rice was narrowed down to a 1.2 Mb region between markers RM3719 and RM7000 for gene identification and also introgression purposes [63]. Biswas et al. (2017) discovered, on chromosomes 6, 8, 11, and 12, six significant QTL for two cold tolerance indices, cold-induced leaf discoloration and improved survival rate following a seven-day recovery period. qCTSL-8-1 and qCTSS-8-1 are co-localized on chromosome 8 at RM7027–RM339, and qCTSL-12-1 and qCTSS-12-1 are co-localized on chromosome 12 at RM247–RM2529 [29]. In order to find stable QTL for cold tolerance at the booting stage, a breeding population made up of 497 advanced lines with Huanghuazhan as the recurrent parent and eight different elite indica lines as the donors was used. The association analysis from this study revealed the QTL qCT-3-2 is consistent for cold tolerance stress across years [64]. Recombinant inbred lines (RILs) produced from a cross between a cold-tolerant variety, Kongyu131, and a cold-sensitive variety, Dongnong422, were employed by Sun et al. (2018) to screen for cold-tolerant loci in rice during the booting stage. In the 28.4 cM interval on chromosome 6, a unique significant QTL, qPSST6, was discovered. Additionally, haplotype analysis shows that *LOC Os06g39750* plays a significant role in controlling rice’s cold tolerance, indicating that it is a candidate gene for qPSST6. Zeta class glutathione S-transferase-encoding candidate genes *OsGSTZ1* and *OsGSTZ2* were eventually identified as candidate genes in a significant QTL, qCTS12 [65]. Using RILs from a hybrid between the cold-tolerant Nipponbare (japonica) and 93-11 (indica), a QTL gene called CHILLING-TOLERANCE DIVERGENCE 1 (*COLD1*) was discovered. This gene is linked to variations in rice cultivars’ resistance to chilling, and it has tremendous promise for rice molecular research [66].

#### 3.1.3. Submergence Stress

SUB1 is a major QTL which is derived from the submergence-tolerant rice FR13A landrace, which has the ability to confer a high degree resistance to flash floods/submergence for 2–3 weeks [67]. A tiny chromosomal area has been narrowed down to identify the genomic fragment encoding the SUB1 QTL. This particular QTL has been fine mapped for identification of three ethylene-responsive factor genes: *SUB1A, SUB1B,* and *SUB1C* [31,68]. The *SUB1* gene discovery in rice, which controls submergence tolerance, was a breakthrough in the history of submergence tolerance breeding. The *SUB1A* gene provides the highest tolerance to submergence [68]. Under waterlogging stress, *SUB1A* inhibits internode elongation and enhances fermentative metabolism. The submergence-tolerant variety FR13A, which has SUB1A, does not expand under waterlogging, but regenerates following water recession, whereas other varieties lacking *SUB1A* grow fast during submergence to avoid the stress [69]. Septiningsih et al. (2012) discovered four unique QTL on chromosomes 1, 2, 9, and 12 in F2:3 populations in a hybrid population of moderately submergence-resistant rice cultivars between IR72 and Madabaru. There are also three non-SUB1 QTL discovered from cultivar IR72, implying that there are other possible paths to the *SUB1* gene’s ethylene-dependent mechanism [70]. The discovery by Xu and Mackill (1996) allowed the SUB1 QTL, which gives 2–3 weeks of submergence tolerance, to be widely exploited in several breeding projects to generate submergence-resistant cultivars in South and South-East Asian nations such as the Philippines, Indonesia, and India. The identification of the SUB1 QTL allows it to be incorporated into high-yielding rice cultivars such as BR11, CR1009, Thadokkam 1 (TDK1), IR64, Samba Mahsuri, and Swarna using marker-assisted backcrossing (MABC) [71].

#### 3.1.4. Salinity Stress

Saltol, a significant QTL linked to rice seedling salt tolerance, was discovered on chromosome 1 by utilizing RILs generated from salt-resistant Pokkali and salt-sensitive IR29 [72]. As a donor in breeding programs, Saltol contributed to improved salt tolerance in several cultivars [73]. Salt stress is reported to be very damaging to rice during the germination and seedling phases [74]. It is most harmful when salinity stress affects rice output during the early seedling stage. Early seedling stage salt stress is very crucial since it determines the grain yields [75]. Lei et al. (2020) reported that rice under salt stress had a significant QTL, qRSL7, on chromosome 7 that influenced relative shoot length (RSL) during the bud burst stage [76]. Zeng et al. (2021) reported the largest effective QTL for salt stress to be qGR6.2, accounting for more than 20% of phenotypic variance for salt tolerance. At the seed germination stage, *LOC Os06g10650* and *LOC Os06g10710* were found to be differentially expressed among five candidate genes with significant transcript abundances. The expression of *LOC Os06g10650* was significantly up-regulated during seed germination under salt stress in two parents. All of this implies that *LOC Os06g10650*, which encodes a tyrosine phosphatase family protein, could be the qGR6.2 candidate gene [77]. The QTL qSE3, that encodes the potassium transporter *OsHAK21* and promotes seed germination under salinity stress by regulating abscisic acid metabolism, was recently found in a *japonica* landrace, boosting seed germination and seedling establishment [78]. *OsMYB6*, on the other hand, is a stress-responsive factor that acts as a positive regulatory element in drought and salt stress resistance in rice [79]. Part of the rice MYB family, *AtMYB111*, regulates flavonoid synthesis and serves as a positive regulator in salt stress reaction. This demonstrate that flavonoids are important for mitigation of salt stress [80].

#### 3.1.5. Metal Toxicity Stress

Although rice is a major source of dietary Cd for persons who eat rice as their main caloric source, Cd can be a poisonous metal [81]. Liu et al. (2019) discovered a shoot Cd accumulation resistance QTL, scc10, and three grain Cd accumulation resistance QTL to overcome Cd toxicity in rice. qCd-2 and qCd-7 are the two QTL that were found in a recombinant inbred population generated from Xiang 743/Katy that was produced in Cd-polluted areas and was utilized in QTL mapping of Cd accumulation in rice grains [82]. Luo et al. (2018) found 13% variance in leaf Cd concentration and cloned Cd accumulation in leaf 1 (CAL1) in a DH rice population. CAL1 controls cadmium transfer from the root to the shoot through the xylem vessels, and CAL1 knockout mutants have drastically lower Cd levels in rice [83]. In another study by Ueno et al. (2009), a high-impact QTL for Cd accumulation in rice was discovered that explained 85.9% of the phenotypic variation in shoot Cd concentration from the Anjana Dhan/Nipponbare population. When overexpressed, OsHMA3, a gene identified in this QTL, can improve rice resistance to Cd and minimize Cd accumulation in grains [84]. Liu et al. (2019) employed 276 accessions containing 416K of single nucleotide polymorphisms (SNPs) to conduct a GWAS on Cd level in rice grain cultivated in severely multi-contaminated farmlands with heavy metals and discovered 22, 17, and 21 QTL relevant for grain arsenic (As), Cd, and Pb concentration, respectively [85].

Fe toxicity in lowland rice may be avoided through the utilization of *Oryza glaberrima* that may provide toxicity resistance genes [86]. QTL mapping in BC3DH lines under Fe^2+^ conditions led to the discovery of 28 QTL on chromosomes 5 and 10 for 11 morphological and physiological characteristics related to Fe toxicity level [87]. Murugaiyan et al. (2019) carried out a study on QTL of As toxicity tolerance and accumulation in rice seedlings between WTR1 (*indica*) and Haoannong (*japonica*). From this study, nine major QTL related to As toxicity were identified. One QTL on relative chlorophyll content in chromosome 1, two QTL for As content in roots on chromosome 8, and six QTL for As content in shoots on chromosomes 2, 5, 6, and 9 were found [88]. Further analysis on these QTL intervals revealed twenty-five genes that exhibit transcription regulation as potential gene candidates for As toxicity traits. Wang et al. (2013) investigated Hg^2+^ tolerance QTL in a recombinant inbred rice population between two japonica cultivars, Yuefu and IRAT109. On chromosomes 1, 2, and 5, three putative QTL were discovered, which contributed around 35.7% of the phenotypic variance in Hg^2+^ tolerance [89]. Table 1 contains a list of QTL linked to abiotic stress tolerance in rice.

According to compiled studies, most of the drought tolerance QTL for rice are found on chromosomes 1, 2, and 3, such as qGY1.1, qDTY2.2, and qDTY3.1. Additionally, there are other major QTL that have been identified in other chromosomes. The major QTL reported, which are qDTY1.1 and qDTY12.1, are associated with grain yield traits. DRO1 is another major QTL identified associated with root trait. This shows that root systems play an important role in rice for drought tolerance. According to researchers, these major QTL have demonstrated consistent effects across two or more genetic backgrounds. This demonstrates the validity and consistency of these QTL, which may be used to improve rice’s drought resistance through plant breeding. Some of the important candidate genes identified for rice in drought tolerance are *NAC*, *OsERF48*, and *DREB*. Even though individual genes have been proven to regulate yield under controlled drought experiments, a well-coordinated response of many genes is essential for drought tolerance under field conditions.

Major QTL for cold stress are qCT-4 (cold tolerance), qLTB3 (seed fertility), qCTSL-8-1, qCTSS-8-1, qCTSL-12-1, qCTSS-12-1 (seedling stage), qCT-3-2 (cold tolerance), and qPSST6 (percent seed set). Based on these major QTL, traits related to seed development are crucial and need to be given importance to develop cold-resistant rice varieties. When the temperature drops below 17 °C, cold stress develops, which results in poor germination and seedling damage [94]. Candidate genes that can play a role in cold tolerance are *LOC Os06g39750* (qPSST6), *OsGSTZ1* (qCTSS-12-1), *OsGSTZ2* (qCTSS-12-1), and *COLD1*. At the rice booting stage, the QTL qPSST6, which is important for the production of long-chain fatty acids, was discovered to be a cold tolerance gene [95]. However, the role of the gene *LOCOs06g39750* in cold tolerance has yet to be explored and needs verification. It is important to explore these genes to improve the understanding of the mechanism of cold tolerance in rice.

SUB1 continues to dominate the submergence breeding studies. The *SUB1* and the *FR13A* genes regulate the submergence resistance against various backgrounds. QTL have been successfully introgressed into the high-yielding variety Swarna using marker-assisted backcrossing (MABC), [96,97]. Submergence-tolerant rice lines BT7 from Vietnam and BR22 from Bangladesh have been improved by using the MABC method to be more adaptable in these submergence-prone regions [98,99]. Three ethylene-responsive factor genes identified in this QTL are SUB1A, SUB1B, and SUB1C. Although fewer major QTL and candidate genes are identified, it is possible to find superior alleles of the SUB1A gene or some newer genes, which may offer better tolerance under submerged conditions.

Major QTL compiled for salinity tolerance or resistance are Saltol, qRSL7, and qGR6.2. The Saltol QTL and Pokkali variety remain the main players in breeding for salinity resistance. *LOC Os06g10650* (qGR6.2), OsHAK21, *OsMYB6* (qSE3), and *AtMYB111* are the main candidate genes that may be utilized for transgenics or breeding. For metal toxicity tolerance in rice, major QTL such as qCd-2, qCd-7, CAL1, qFeTox4.3, qFeTox6.1, qFeTox10.1, qGAS1, qAsS2, qRChlo1, and qRRL-1 are crucial depending on the metal toxicity faced. Despite the number of studies conducted, one major gene that is mentioned consistently is *OsHMA3* for improved rice resistance to Cd.

### 3.2. Major Abiotic Stress QTL and Candidate Genes in Maize

Maize (*Zea mays* L.) has been the most productive cereal crop since its global spread and is of immense significance for human consumption and use [100] as basic raw material for production of starch, protein, oil, alcoholic beverages, food sweeteners, and fuels [7]. Hence, there is a strong need to develop well-characterized maize cultivars that can survive high degrees of abiotic stress and perform well under these conditions via the development of new and enhanced varieties.

#### 3.2.1. Drought Stress

Anthesis-silking interval, which reflects plant susceptibility to abiotic stress and significantly correlates with grain yield, is an excellent secondary feature for drought tolerance in maize [101]. Zhao et al. (2019) reported 20 stable QTL for yield and growth traits under different water stress scenarios in maize [102]. In the same year, Abdelghany et al. (2019) discovered 167 QTL for ear length, diameter, weight, kernel weight per ear, and hundred-kernel weight under six drought stress conditions using 213 hybrid families of H082183 (drought tolerant) and Lv28. A total of 48 QTL were discovered, with 15 of them linked to nine characteristics with substantial QTL-by-environment interactions [103]. Crown root angle (CRA2) and crown root length (CRL1) are two QTL with antagonistic pleiotropic (control several traits) effects on access to water [104]. In another study on vigor and stay green traits under drought conditions, chromosomes 1 and 2 showed the presence of three significant QTL for the anthesis-silking interval, plant height, and senescence [105]. Almeida et al. (2014) studied three tropical bi-parental populations in Mexico, Kenya, and Zimbabwe under water stress and well-watered conditions to discover regions of the maize genome responsible for grain yield and anthesis-silking interval across varied habitats and genetic backgrounds. In all three populations, one QTL on chromosome 7 for grain yield and one on chromosome 3 for anthesis-silking interval were shown to be ‘adaptive’ to water-stressed environments [19]. Based on GWAS analysis for drought tolerance in maize seedlings, four genes were linked with malondialdehyde activity, three genes linked with superoxide dismutase activity, and one gene to relative conductivity [106]. In a study of 368 maize varieties gathered from tropical and temperate regions, Liu et al. (2013) assessed all of the functional dehydration-responsive element binding (*DREB*) protein genes and looked at their connections with spontaneous variation in drought resistance. Drought tolerance and natural variation of ZmDREB2.7 in the promoter region were shown to be significantly correlated. The variable levels of drought tolerance among maize varieties were linked to DNA polymorphisms in the ZmDREB2.7 promoter region but not in the protein-coding region itself. This association is probably caused by different patterns of gene expression in response to drought stress [107].

#### 3.2.2. Cold Stress

One of the primary challenges restricting maize yield in mid–high-latitude parts of the world is cold stress. Allam et al. (2016) identified 27 QTL for cold tolerance in B73 × P39 and 24 QTL in B73 × IL14 h maize populations where these QTL were dispersed across all chromosomes except chromosome 10. There are two major QTL for the traits vigor and ear height on chromosomes 4 and 8, respectively, with phenotypic variation of 37.8% and 43.3% for cold tolerance in maize [108]. Based on GWAS analysis in a study on 282 maize lines of the inter-mated B73 × Mo17 (IBM) Syn4 RIL, six QTL on chromosomes 4, 5, 6, 7, and 9 were shown to be associated with low-temperature germination and root length. For low-temperature germination rate, qLTGR5-1 had the most significant LOD values and contributed the most to phenotypic variance, whereas for the root length trait, qLT-PRL9-1 had the highest LOD values and explained the majority of the phenotypic variation [109]. Han et al. (2022) reported seven QTL that formed five QTL clusters on chromosomes 1, 2, 3, 4, and 9 for germination traits under a low-temperature environment, suggesting that some genes may be regulating several germination features simultaneously. By combining the study of RNA-seq and QTL-located genes, three up-regulated B73 genes and five up-regulated Mo17 genes were discovered. It was suggested that the difference in low-temperature germination tolerance between B73 and Mo17 might be due to the expression levels and amino acid sequence variation of candidate genes. Male sterility is also induced during the reproductive period due to low temperatures, which reduces the production of important cereals. To date, qCTR5 and qCTR12 on chromosomes 5 and 12 of the maize genome have been identified as being influenced by this characteristic throughout the reproductive phase. Two candidate genes for qCTR5, O-methyltransferase ZRP4 and beta-1,3-glucanase-like protein, and one for qCTR12, a conserved putative protein, were established by gene expression analysis [110]. A study by Jin et al. (2021) employed two methods, (i) GWAS and (ii) QTL mapping of two populations, to identify QTL related to cold tolerance in maize. The QTL qPOD3 was identified as a major QTL for cold tolerance in maize using a first population (80 inbred lines) and second population (W72 × W10). This QTL is associated with the gene Zm00001d002729. The presence of Zm00001d002729 in both populations has substantially proved the gene’s reliability, resulting in greater cold tolerance [111]. In a 176 IBM Syn10 doubled haploid population from the B73 Mo17 cross, five QTL clusters were possibly linked to low-temperature germination-related phenotypes. These clusters were made up of seven QTL that were situated on chromosomes 1, 2, 3, 4, and 9. There are 39 candidate genes in these five QTL clusters [112]. Twelve articles looked into more than two cold tolerance indicators. Eleven of these studies employed several populations as the experimental material for the cold tolerance gene mining rather than a few inbred lines or hybrids [113].

#### 3.2.3. Submergence Stress

A study on the effect of submergence on maize identified QTL on chromosomes 4 and 9 for root/shoot dry and fresh weight that explained 6.3–12.0% and 30% phenotypic variance under waterlogged circumstances, respectively. However, most major QTL are located on chromosome 9 and were consistently identified in both experiments in this study [114]. Subtol6, a QTL related to submergence tolerance in maize, has been identified on chromosome 6 which explains 22% of the phenotypic variation. Subtol6 has two underlying candidate genes, *HEMOGLOBIN2* and *RAV1* [115]. Both genes regulate Arabidopsis leaf senescence and limit ROS levels in maize. Yu et al. (2019) reported that waterlogging tolerance is aided by *ZmERB180*, which belongs to the group of VII ethylene response factors in maize seedlings [116]. *GRMZM2G055704*, a heavy metal transport protein, was revealed as a waterlogging resistance candidate gene by implementing bulked segregant RNA-seq (BSR-seq) in 10 susceptible and eight tolerant inbred lines in maize. In the tolerant line (CML495), *GRMZM2G055*704 was highly up-regulated, whereas in the sensitive line (CML495), it remained significantly down-regulated (CMTL001) [117].

#### 3.2.4. Salinity Stress

Maize is susceptible to salinity stress during germination and seedling growth, which results in poor kernel set and lower grain weight and quantity, hence reducing total production [118]. Among the traits related to salinity stress that were consistently studied are plant growth and root-related traits. A major QTL on chromosome 1, qSPH1, contributed significantly to salt tolerance-related phenotypes, explaining 25.9–31.2% of the phenotypic variance [119]. In another study, 209 DH lines produced from the maize hybrid Xianyu335 were genotyped using 1335 SNP markers. QTL analysis for salt tolerance was conducted using biomass-related traits during salt stress in a hydroponic culture under normal and salt-stressed conditions. Salt tolerance-related QTL were found on chromosomes 1, 3, 7, and 9. Among these chromosomes, 13 QTL on chromosome 1 contributed 21% of the phenotypic variance. A few genes linked to salt tolerance in maize have also been discovered and described. Some of these genes were transcription factors, such as *ZmbZIP72*, *Zmhdz10*, and *ZmWRKY58*, whereas others were protein kinase genes such as *ZmSIMK1*, *SnRK2*, and *ZmSnRK2.11* [120]. In maize, *ZmHKT1* encodes an HKT type transporter, and has been identified from the salt tolerance QTL ZmNC1. *ZmHKT1* is required for Na^+^ homeostasis and salt tolerance in maize [121].

#### 3.2.5. Heavy Metal Toxicity

QTL mapping was used to investigate the genetic basis of arsenic (As) build-up in a recombinant inbred population originating from the Chinese crossbred variety Yuyu22. Twenty-eight (28) QTL associated with As concentration in various maize tissues were discovered from two locations, Xixian and Changge, in northern China. In a combined analysis of the two locations, 11 QTL were discovered out of 28. At Xixian, the chromosome 1 gene XAsK1a explained a significant percentage of the variance in kernel As concentrations (26.50%) [122]. Meanwhile a GWAS was conducted in a population of 269 maize accessions containing 43,737 SNPs to discover potential genes and favorable alleles for regulating Cd accumulation in maize. A major QTL on chromosome 2, qLCd2, with 39.8% average phenotypic variation, was found through a GWAS and validated by QTL mapping with the IBMSyn10 DH population. Expression of candidate gene *GRMZM2G175576* was identified in response to the Cd stress QTL qLCd2. This gene codes for a cadmium/zinc-transporting ATPase and was increased significantly in the roots, stems, and leaves of the B73 maize line [123]. QTL for Pb and Cd toxicity were identified in maize using the IBM Syn10 DH population, where 42 QTL were discovered using the Pb and Cd tolerance coefficient. Two major QTL responsible for the combined heavy metal tolerance were identified among these QTL. Four potential genes, *Zm00001d048759, Zm00001d004689, Zm00001d033527*, and *Zm00001d004843*, within these major QTL, were associated with heavy metal transport and tolerance [124]. Table 2 below lists QTL linked to abiotic stress tolerance in maize.

For drought stress tolerance in maize, two major QTL were identified based on the studies compiled for traits such as crown root angle, CRA2, and crown root length, CRL1. It is observed that root traits play a crucial role in maintaining maize yield under water stress conditions because they impact the amount of water absorbed. Due to increased climatic variability under the present agricultural methods, the high permeability of root characteristics becomes even more crucial in water- and nutrient-deficient soil [125]. Malondialdehyde, superoxide dismutase, and *DREB* genes play a crucial role in drought management in maize.

Various major QTL with traits such as root development, growth, germination, and seedling development are reported. However, seed germination and early seedling development are the two stages that are most sensitive to low-temperature stress. Hence, focusing on identifying major QTL related to these traits will effectively produce higher yield for maize in cold climate conditions. *Beta-1,3-glucanase-like protein* and *O-methyltransferase ZRP4* are potential genes for further study in low-temperature response regulation and resistance. QTL such as Subtol6 and those identified on chromosome 9 were identified as important QTL for waterlogging tolerance in maize. Candidate genes such as *HEMOGLOBIN2, RAV1, ZmERB180*, and *GRMZM2G055704* were identified within these target QTL. With climate change anticipated to bring more frequent floods, major QTL and genes may help to boost survival rates in maize. Very few gene variants have been verified from these studies for maize submergence tolerance. It is possible that reliable genes that control submergence tolerance are hard to discover due to the complicated regulatory network and the variety of morphological and metabolic responses.

Major salinity stress QTL were identified predominantly on chromosomes 1 and 3 (qSPH1, qRLS1, qSLS1-2, qFLS1-2, qRFS1, qFFS1, qRLR1, and ZmNC1). Potential candidate genes identified as regulating salinity stress were HKT type transporter and protein kinase. These genes are crucial in transmembrane signaling and transport which are essential in salt stress management and are good targets for introgression to create salt-tolerant maize cultivars Likewise, several QTL and genes were identified for heavy metal tolerance. These discoveries will aid in identifying functional genes and QTL for molecular marker-assisted breeding for heavy metal resistance. This is important with the chemical-heavy agricultural practices leading to heavy metal content in the soil, and impacts on soil health and fertility.

Although many QTL have been detected via linkage mapping, few studies report on the fine mapping of QTL that enables the identification of the precise genetic position and/or the cloning of candidate gene(s). This is because large secondary populations are generally required to achieve sufficient map resolution, which requires a high level of resources and are time-consuming to establish. The large amounts of repetitive sequences in the maize genome have hindered progress in QTL fine mapping and cloning.

### 3.3. Major Abiotic Stress QTL and Candidate Genes in Wheat

Wheat (*Triticum aestivum* L.) is a significant crop that contributes approximately 20% of the calories consumed by humans globally. Wheat output has increased due to current genetic and genomic enhancements; nevertheless, more improvements are required to feed the world’s population, which is expected to reach over nine billion by 2050 [126]. More than half of the world’s durum wheat is cultivated in the Mediterranean basin, but it is also grown in a smaller amounts in the northern plains of the United States and Canada, the desert areas of the southeast United States and northern Mexico, and other locations [127].

#### 3.3.1. Drought and Heat Stress

Grain yield is a crucial feature that needs to be enhanced in wheat. However, because it is impacted by the environment, it is influenced by genotype–environment interaction, leading to poor heritability. In addition, all 21 of the wheat chromosomes include grain yield-related QTL. These characteristics make it challenging to analyze the genetic architecture of this trait [128]. Despite this, several genetic studies on the genetics of grain yield, including QTL analyses, have been carried out. Dolferus et al. (2019) employed a Cranbrook × Halberd DH population where QTL for spike grain under drought stress were identified on chromosomes 5A and 2A in wheat [21]. Further, Liu et al. (2019) reported 71 QTL, of which eight were common among heat, drought, and heat and drought stresses in wheat. Five QTL hotspots for yield and related characteristics were found in chromosomes 2A, 3D, 6D (two), and 7B under all the above stressors. The parental line in this study, SYN-D, provided 37 QTL, and the rest were provided by Weebill 1 [24].

In a recent multi-location study on wheat, a main effect genomic region for yield, QYld.aww-1B.2, was finely mapped to a 2.9 cM area that correlates with 39 predicted genes. This finely mapped QTL may be readily targeted for introgression studies [129]. Another study by Gautam et al. (2021) reported the introgression of a major drought tolerance QTL for yield, Qyld.csdh.7AL, into elite wheat cultivars, namely HUW234, HUW468, K307, and DBW17, which exhibited a low stress sensitivity index and were verified by their higher yields when grown in a rain-fed environment [130]. Early vigor, leaf area, and root architecture are a few factors that have been linked to yield in wheat [131]. Through a GWAS, a major QTL, qSRA-6A, was identified for seminal root angle. This particular QTL is reported to have potential in root architecture characteristics in cultivars and can improve crop stability in areas with little rainfall [132]. In a study by Maccaferri et al. (2016) in durum wheat, three significant QTL for the seminal root angle were identified in two contrasting mapping populations and were suggested for further study as causal genes related to drought tolerance [133]. In a more current study for wheat, 11 consistent and stable QTL for traits related to drought tolerance were reported in the drought-tolerant cultivar ‘Reeder’ and the high-yielding cultivar ‘Albany’. Six of these QTL were identified in drought environments and another five were identified in constitutive conditions (drought and normal environments) [134]. NAC transcription factors, protein-containing kinase domain, homeobox domain proteins, and HSP70 were previously shown to have a function in drought stress in wheat [135].

#### 3.3.2. Cold Stress

In common wheat, major loci controlling freezing tolerance have been identified on the long arm of group 5 chromosomes [136]. To date, only two significant wheat QTL for cold tolerance have been found on chromosome 5A, which are Frost Resistance-1 (Fr-1) and Frost Resistance-A2 (Fr-A2) [27,137]. The major frost tolerance locus, Fr-1, was discovered 2 cM from the vernalization gene *Vrn-A1* on the long arm of chromosome 5A. According to QTL mapping, frost tolerance and vernalization requirements are regulated by two closely linked loci on chromosome 5A, *Vrn1* and *Fr1*. Physical mapping with Chinese Spring deletion lines indicates that *Vrn-A1* and *Fr-A1* are distinct genes with strong interactions between *VRN1* and *FR-A2* for frost tolerance in both winter and spring panels [136]. An additional frost tolerance gene, *Fr2*, has been discovered on chromosome 5D in the homoeologous region matching the one harboring *Fr1* on chromosome 5A, indicating that these two QTL are orthologous [138]. Fowler et al. (2016) identified 13 QTL for three low-temperature tolerance characteristics in three wheat populations genotyped with 90K SNP iSelect wheat array. A major grain protein content and yield QTL for both characteristics was discovered in the same region on chromosome 6A while two QTL on chromosome 5A had a major influence on phenological development and low-temperature tolerance [139].

Using recombinant inbred lines of common wheat, QTL analysis of five cold-responsive genes revealed two major freezing tolerance loci. A major QTL, Qct5a, on chromosome 5A that correlates to Fr-A2, was discovered in winter wheat by an association scan and is likely driven by copy number variation of the gene *CBF-A14* found at this location [137]. Cold stress signaling may be associated with many messenger molecules, protein kinases and phosphatases, and transcription factors [140]. Several regulators, such as ICE transcription factors and protein kinases, control the production of C-repeat binding factors (CBFs) and their protein stability (either favorably or negatively) [141]. *ICE* genes, which are MYC-type *bHLH* transcription factors, can induce CBF expression when exposed to cold [142]. Under cold stress, CBFs and other cold-induced transcription factors such as MRKY, ethylene-responsive transcription factor, and heat shock transcription factor can promote *COR* gene expression [140]. In a study by Li et al. (2015), the RNA-seq results revealed that the candidate gene *TraesCS5A01G307000* was elevated in wheat, with increased expression found during low-temperature stress. *TraesCS5A01G307000* encodes a pentatricopeptide repeat-containing protein, which is an RNA binding protein localized to chloroplasts or mitochondria and is probably involved in RNA processing. This gene’s expression pattern implies it is involved in low-temperature stress responses [143].

#### 3.3.3. Submergence Stress

In the wheat W7984/Opata85 population, 32 QTL were found to be associated with waterlogging tolerance parameters, such as survival rate, germination rate index, leaf chlorophyll content, plant height index, and dry matter weight. A major QTL on chromosome 7A explained 23.92% of the phenotypic variation for the germination rate index [144]. Mapping studies in wheat RILs produced from USG3209 × Jaypee discovered 48 QTL clusters in ten chromosomal areas. Under waterlogged field and greenhouse settings, three QTL on chromosome 1BL were discovered. Under controlled greenhouse conditions, another significant QTL (QSpad3.ua-1D.5) on chromosome 1D for chlorophyll content explained 24% of phenotypic variance [145]. In another report by Wei et al. (2019), different gene expression patterns were found in *TaERFVII.1* between waterlogging-tolerant and -susceptible wheat cultivars. The expression of waterlogging-responsive genes was affected in *TaERFVII*.1 silencing lines. Constitutive expression of stabilized *TaERFVII.1* with MYC-peptide tagged at its N terminus improves wheat’s waterlogging tolerance by increasing survival rate and leaf chlorophyll content and inducing waterlogging tolerance-related genes [146].

#### 3.3.4. Salinity Stress

Research on the genetics of wheat’s ability to withstand salinity has received high priority in many nations, including India, Pakistan, Bangladesh, China, Egypt, etc. [128]. Devi et al. (2019) found two QTL under salinity tolerance that were stable in all environments, with explained phenotypic variation ranging from 2.6% to 15.1%. Three SSR markers, gwm 261, cfd 84, and wmc 112, were found to be closely connected to the QTL for K^+^ content, days to heading, days to anthesis, number of tillers, and number of ear heads, respectively. Most of the salt tolerance QTL discovered in the study were derived from cultivar KH65, implying that this cultivar had many salt tolerance genes [147]. In another study, a population from a hybrid between the low Na^+^ landrace and the cultivar Tamaroi was assessed for QTL for low Na^+^ concentration in the leaf blade. Here, the Nax1 locus (Na^+^ exclusion), was discovered on chromosome 2AL, accounting for around 38% of the phenotypic variation in the mapping population [148]. In another study including Na^+^ exclusion, a total of 154 wheat F2 lines were genotyped from a hybrid between salt-resistant and salt-susceptible cultivars. Six of the 49 identified QTL were for Na^+^ exclusion (NAX), and two of the QTL on chromosome 2A (qSNAX.2 A.1, qSNAX.2 A.2) matched the previously reported major QTL, NAX (Nax1 or HKT1,4). On chromosome 7A, two more significant NAX QTL that contribute 11.23 and 18.79% of salt tolerance, respectively, were mapped [149]. Pal et al. (2021) further reported that, through a meta-analysis of salt tolerance QTL, 81 potential candidate genes were found in high-confidence MQTL. These candidate genes encoded proteins such as: F-box protein, pentatricopeptide repeat, phospholipid/glycerol acyltransferase, auxin-up RNA, UDP-glucosyltransferase, glutathione S-transferase protein, B-box-type zinc finger, and cytochrome P450 protein [150].

#### 3.3.5. Metal Toxicity Stress

In metal toxicity stress, it was reported that wheat cultivars, such as Parwaz-94, Kaghan-93, and Auqab-00, were highly resistant to chromium (Cr) stress while maintaining high grain yield. Meanwhile, Sarsabaz, Chenab-00, Iqbal-00, Local white, Shahkar-13, and Pirsabak-05 wheat cultivars showed vulnerability to Cr stress [151]. In durum wheat cultivars, a SNP marker, IWA1775 on chromosome 5BL, was linked to grain Cd content [152]. SNP markers were used to identify a significant QTL, QCdu.ndsu-5B, for Cd absorption on chromosome arm 5BL in durum wheat [153]. Qiao et al. (2021) conducted a study on 181 DH lines of spring wheat for Cd toxicity, wherein three main QTL were found on chromosomes 5B, 7B, and 7D, namely QCd.uia2-5B, QCd.uia2-7B, and QCd.uia2-7D. Three genes from these major QTL, *TaHMA3, TaHMA2*, and *TaMSRMK3*, were identified that regulate the uptake and transport of Cd in wheat [154]. There have been several mapping investigations for Al tolerance in wheat under controlled climatic conditions. On chromosome arm 4DL, a major Al tolerance QTL related to the SSR loci Xgdm125 and Xgwm976 was discovered, explaining 31% of the population’s phenotypic variation. A DH population was employed to map chromosomal arm 3BL for a second major Al tolerance QTL, Qalt CS.ipk-3B, with 49% of the phenotypic variance explained [155]. Schnurbusch et al. (2007) identified a major boron (B) toxicity tolerance gene in wheat, which is *Bo1*. This gene is acknowledged for having a significant role in yield output in cultivars that thrive in B-toxic areas of southern Australia [156].

Compared to rice and maize, wheat has fewer studies on identifying QTL and its candidate genes for abiotic stress. This is due to the large wheat genome size and lack of complete sequence information. However, there may be a way to get around these restrictions in identifying QTL in wheat by combining modern technologies with conventional breeding techniques. These latest studies are encouraging, and together with current developments in DNA sequencing technology, these data will find their way into useful projects for abiotic stress-related wheat breeding. For drought stress tolerance in wheat, most of the major QTL are found for root and yield traits. This proves that root system architecture features are the main target to be improved for breeding of wheat varieties that are drought tolerant. A number of the QTL reported in Table 3 are associated with different traits contributing to wheat grain yield under drought. Among the genes that play a role in drought regulation in wheat are NAC transcription factors, protein-containing kinase domain, homeobox domain proteins, and HSP70. For cold stress tolerance, major QTLs were identified on chromosome 5A and 6A. Key genes such as *ICE* genes, which are MYC-type *bHLH* transcription factors, will help to understand the molecular mechanisms controlling wheat response to cold stress and are potential candidates for development as markers for identification of cold tolerance QTL.

Submergence stress tolerance identified major QTL linked to germination and chlorophyll content. The gene that can be explored is *TaERFVII.1.* Both these processes have to be well regulated under submergence stress for optimal survival. Likewise, for salinity stress, several QTL have been located on chromosome 2A. This region has been mapped with several stress-related proteins such as F-box protein, acyltransferases, auxin-up RNA, UDP-glucosyltransferases, glutathione S-transferases, and cytochrome P450. Finally, heavy metal stress tolerance several QTL specific to metals were identified on several chromosomes. The identified genes have great potential in breeding. Overall, relatively few QTL have been identified for abiotic stress tolerance in wheat. The information provided by every expression array experiment becomes increasingly trustworthy when more gene and protein sequences are made publicly accessible in wheat databases.

## 4. Application of QTL in Cereal Breeding

### 4.1. Stability of QTL across Diverse Backgrounds (Multi-Environment/Multi-Trait)

Despite a large number of studies on QTL mapping for complex traits in key cereal crops over the last decade, introgression of such QTL has been limited, and the number of causal genes found inside these QTL regions remains small. Due to this, it is still difficult to find major stable QTL with closely related markers that have the potential for molecular breeding [157]. This is because most genetic maps have excessively large average marker distances or markers are only dispersed in partial chromosome regions [158]. Furthermore, quantitative traits are highly sensitive to environmental influences, making it difficult to precisely choose target features in the field. As a result, finding resistant genetic resources has become challenging. Further, in these QTL intervals, only a few functional genes have been isolated, cloned, and studied [159]. Thus, stable and consistent QTL across various conditions and genetic backgrounds must be identified and validated [49]. One of the methods suggested for identifying consistent and stable QTL is the division of the population into two groups, one with expression of extreme phenotypes and the other expressing intermediate phenotypes. An extreme sampling of 10 to 35% on each side of the phenotypic distribution curve has previously been shown to be beneficial in discovering linked QTL [160]. Zhang et al. (2021) recommended high-density genetic maps to understand the genetic basis for important traits of interest and to build new DNA-based diagnostic tools or MAS-based breeding approaches. The lifespan of new high-yielding cultivars can be extended by generating varieties with lasting resistance through pyramiding several resistance genes/QTL or employing broad-spectrum resistance genes [161].

Yadav et al. (2019) suggested that the ideal technique in the breeding programs is the detection of grain yield trait QTL that have a major and consistent influence throughout various genetic backgrounds and conditions for drought tolerance. This is because the loci that go through genetic dissection, which influence trait tolerance in rice, will speed up the production of novel rice cultivars with increased grain yield under stress conditions [49]. The discovery of stable and robust QTL in cereals for yield under various stress conditions is essential for preserving cereal adaptation and production stability in the face of changing climate [162]. The positive interaction among QTL against various backgrounds of different popular varieties can contribute towards identifying stable QTL and the QTL with combinations of several abiotic stress tolerances [163]. From our standpoint, it is suggested to carry out assessment trials often in many environments, ideally with contrasting environmental conditions, and over many years, involving many critical attributes for cereal growth and high yield. Genotype-by-environment interaction (GEI) has also been reported to be important for improving root characteristics across different environments [164]. In a recent study, Oo et al. (2021) successfully introgressed qDTY12.1 into Pusa 44 through MABC, resulting in the generation of enhanced NILs with improved reproductive-stage drought tolerance adaptability in two different environments. Their discovery across a wide range of genetic backgrounds and/or environments suggests that such QTL alleles could be effective in MAB due to consistency across different genetic backgrounds [165]. Besides the multi-environment interaction, the gene-for-gene interactions are also a source of unexplained genetic differences in complex traits. However, these interactions are generally neglected in GWASs and other methods of genetic dissection of QTL.

### 4.2. Linkage Mapping vs. Association Mapping

Linkage mapping and association mapping are two popular and successful techniques for finding novel genes associated with specific traits and have been utilized with great success in cereal species [166]. Compared to association analysis, linkage analysis is more accurate for studying species with low genetic variation. Both linkage mapping and association analysis can be used in a cross-validation combination of complimentary procedures [167]. However, association mapping is more commonly used to map traits, such as grain yield, grain quality, flowering and grain production features, high-temperature stress, drought tolerance traits, and salinity tolerance traits [168]. Linkage mapping has a low accuracy and high power, whereas association mapping has a high resolution. Compared to traditional linkage mapping, there are three advantages of association mapping. Firstly, (i) it decreases the time and cost of developing suitable segregating populations, and it allows for a wider range of information by utilizing existing populations; (ii) it can detect several allelic variations and recognize favorable alleles connected to a target trait in a single analysis; and (iii) the fine mapping of QTL is aided by its high resolution [169,170]. Despite their differences, when linkage and association mapping approaches are integrated, they provide an excellent method for identifying QTL and molecular markers for rapid breeding deployment.

### 4.3. Conventional Cereal Breeding

Conventional breeding (traditional breeding) involves the generation of new plant varieties using older methods and natural processes [171]. Since the 1990s, molecular markers have been used to identify superior hybrids by pyramiding various resistance genes and generating multi-line cultivars with sustainable resistance to abiotic stresses [172]. SSRs and SNPs detect DNA variations across closely related populations and can even identify single nucleotide alterations at the whole genome level [173]. Microsatellites or SSR markers are the most well-known polymerase chain reaction (PCR)-based markers. These markers are widely used among cereal species for screening, characterizing, and assessing genetic variation in a variety of cereal species because they are co-dominant, hypervariable, locus sensitive, and multi-allelic [174]. Due to a high level of co-dominant and allelic variation characteristics, SSRs are recognized as the ideal markers for building genetic linkage maps and analyzing QTL and have been used extensively in cereal breeding [174,175].

On the other hand, the introduction of NGS and high-throughput genotyping technology has made it relatively easy to detect and use SNPs [176]. SNP marker technology allows wheat breeding programs to use low-cost, easy-to-use molecular markers for MAS. Furthermore, it is the most common form of marker in cereals [177,178]. SNPs are also becoming suitable automated genotyping tests with high throughput, allowing samples to be genotyped faster, more efficiently, and with lower cost than SSRs. The use of high-density SNP iSelect assays (9K and 90K) in *T. aestivum* has contributed a large number of markers to detect QTL with economically important traits and the identification of genomic areas targeted for breeding programs [179]. Genotyping by sequencing (GBS) is a genome-wide yet limited representation method that produces a huge number of sequence variants from a big population. GBS was created for high-resolution association studies in maize and has now been used in a variety of species with complex genomes. GBS has been optimized in many crops, including maize, wheat, barley, rice, potato, and cassava, for efficient, low-cost genome sequencing at large scales [180]. Bhattarai and Subudhi (2018) employed a saturated linkage map based on GBS to find drought-sensitive QTL during vegetative development [181]. NGS technology has lowered the cost of DNA sequencing to the point that GBS may now be used for routine breeding screening in any crop [182]. The GBS method is appropriate for large and complex genomes, such as wheat genotypes, because it uses two enzymes to reduce genome complexity by avoiding repeated sections in large genomes [183].

Phenotyping efficiency restrictions are often seen as major roadblocks to genetic improvements in breeding operations [184]. In traditional breeding, MAS, or genomic selection, the technique of high-throughput phenotyping (HTP) may induce a bottleneck as phenotyping is required to verify the reliability of statistical models. To eliminate undesirable phenotypic combinations, backcrossing the selected offspring with the recipient line for several generations can also impart desirable characteristics into a chosen ‘best’ recipient line [185]. Although conventional plant breeding has a long history of improving crop productivity, food security, and safety, it is inadequate and poses certain challenges in cereal genome enhancement. Hence, it is crucial to explore new techniques and breeding methods that can be applied along with conventional plant breeding methods to develop cereals that are resistant to abiotic stress.

### 4.4. Mutational Breeding

Mutation breeding is another way of improving cereal varieties through conventional breeding. Mutagenesis is a phenomenon in which an organism’s genetic material undergoes abrupt heritable alterations. It can happen naturally or in response to exposure to various biological, chemical, and/or physical stimuli. The three categories of mutagenesis are used to classify mutation breeding. The first is radiation-induced mutagenesis, which is caused by exposure to radiation such as gamma rays, ion beams, and X-rays [186]. The use of gamma radiation from radioactive cobalt is common. It is dangerous and has a high penetrating potential. However, it can be used to irradiate entire plants and delicate materials such as pollen grains [186]. Considering gamma rays have shorter wavelengths, they contain more energy than protons and X-rays, allowing them to penetrate deeper into a tissue [187]. Ion beams, created by particle accelerators, have fast velocity (between 20% and 80% of the speed of light) to produce high linear energy transfer (LET) radiation. Compared to other types of radiation employed in physical mutagenesis, the damage generated by ion beams in DNA double strands is less repairable than the damage caused by gamma rays due to the deletion of DNA fragments of various sizes [188]. Physical mutagenesis has a significant advantage over chemical mutagenesis for precision and reproducibility, especially for gamma rays, which have consistent penetration strength in tissue [186]. The second is chemical mutagenesis through nucleotide substitutions in the DNA, changing the amino acid sequence, which further modifies the way proteins function [189]. Only a small number of alkylating chemicals have been used extensively in plant experimental mutagenesis and plant mutation breeding. Three chemicals are particularly important: ethyl methane sulfonate (EMS), 1-methyl-1-nitrosourea, and 1-ethyl-1-nitrosourea, which account for 64% of mutant variants [186]. Chemical mutagens are known to have less effect on plant materials than other mutagens. Chemical mutagenic agents have the advantage of not requiring complicated equipment or facilities [190]. The third is insertion mutagenesis, which occurs due to DNA insertions, either via transformation of plant genetic and T-DNA insertion or transposable element activation [15]. T-DNA insertions, which can cause loss of function as a direct response to their biological function, are the most extensively utilized techniques for gene function identification [191]. By improving cereals’ tolerance to abiotic stress, the latest mutant breeding methods and emerging breeding tools expand their potential for use in addressing food security concerns.

### 4.5. Marker-Assisted Selection (MAS)

MAS is a relatively new emerging method of improving phenotypic selection criteria by selecting genes, indirectly or directly, as an alternative to traditional breeding [192]. However, the efficiency of MAS depends on identifying the accurate location of QTL and tightly linked molecular markers. Therefore, the combination of MAS with traditional phenotypic selection can increase breeding efficiency and improve the precise transition of target alleles into the advanced progenies in a shorter time [172,193].

Marker-assisted foreground selection and background selection have proven to be beneficial for breeding significant gene-controlled traits. Two prominent MAS schemes, marker-assisted recurrent selection (MARS) and genomic selection (GS), are useful for complex features/traits that are often controlled by QTL with minor effects [194]. Identifying markers linked to QTL has been the focus of MARS for quantitative traits. MARS is important for improving bi-parental populations’ stress tolerance because it harnesses many QTL containing the most desired combinations of favorable alleles and uses only significant markers to predict population performance [195]. Bankole et al. (2017) proposed using MARS to generate drought-resistant inbred lines, based on the majority of small impact QTL for drought tolerance [196]. Later, Cerrudo et al. (2018) recommended using QTL-MAS in forward breeding to accumulate desirable alleles that have strong additive-effect QTL in early selection cycles. Identifying the QTL that underpin these genes will aid in developing more precise DNA markers that are gene specific for MAS, as well as understanding the physiological and genetic underpinnings of abiotic stress in cereals. Such studies would allow for the development of new and stronger alleles for abiotic stress tolerance [197].

MAS 946-1 was the first drought-tolerant aerobic rice created using MAS technology [198]. Barik et al. (2019) discovered five QTL related to relative water content, leaf rolling and drying, and spikelet fertility from a mapping population derived from a hybrid between CR 143-2-2 and Krishnahamsa. Out of the five QT, four were unique and should be useful in the MAS strategy to develop drought-tolerant rice [199]. Based on gene pyramiding of a Malaysian rice under reproductive drought stress, Shamsudin et al. (2016) found that three drought-related QTL, qDTY2.2, qDTY3.1, and qDTY12.1, consistently affected grain yield and these three QTL were successful in the initial selection in each of their breeding generations [200]. Meanwhile, Mujtaba et al. (2018) measured the potential for desiccation resistance in 26 wheat genotypes under drought stress. They discovered six highly drought-tolerant genotypes (MAS-2/2014, MAS-3/2014, MAS-8/2014, MAS-12/2014, MAS-18/2014, and MAS-20/2014), perfect for boosting rainfed and dry area production [201]. Further, Gautam et al. (2021) inserted a yield-related QTL, Qyld.csdh.7AL, into four wheat cultivars, HUW468, HUW234, DBW17, and K307, to produce a high-yielding drought-tolerant genotype [130].

Pyramiding is another process of combining numerous genes or QTL into a single genotype simultaneously [202]. Anyaoha et al. (2019) pyramided the FUNAABOR-2 rice variety with two QTL, qDTY12.1 and qDTY2.3, using the marker-assisted gene pyramiding (MAGP) method. The pyramided lines in the resultant rice variety had greater yields than the lines with a single QTL or no QTL, indicating that pyramided QTL had strong positive interactions between them to transmit drought resistance genes during the reproductive stage [203]. Muthu et al. (2020), for example, created a multiple stress-tolerant variant to improve White Ponni by pyramiding key effect QTL, such as qDTY1.1 and qDTY2.1 for drought tolerance, Saltol for salinity tolerance, and Sub1 for submergence tolerance. The co-location of drought tolerance QTL for grain yield, stay green, and ears of cereals on chromosome 1 also validated the physiological link and high correlation between these characteristics [204]. The presence of these traits in the same region might imply that this region could be a hotspot for yield-related traits and that introducing this region into maize genotypes will result in high-yielding varieties. The efficiency and use of MAS for pyramiding genes in wheat were explored for the possibility to pyramid up to 12 genes/QTL in wheat. By applying the above, enhanced wheat lines with amber grains that were genetically modified to have genes for grain quality, grain weight, and rust tolerance were created. MAS was used to create eight pairs of NILs for grain weight by transferring three wheat QTL identified from an earlier study for grain weight (QGw.ccsu-1A.3, QGw.ccsu-1A.2, and QGw. ccsu-1B.1) to validate the effect of the three QTL on grain weight in wheat [205].

By combining marker-assisted recurrent selection (MARS) and marker-assisted selection (MAS), Sandhu et al. (2018) created two mapping populations by crossing drought-tolerant donor IR 87728-75-B-B with drought-susceptible Samba, which possessed qDTY1.1, qDTY2.1, qDTY3.1, and qDTY11.1 [163]. Studies have identified three quantitative trait loci for grain yield under drought conditions, qDTY 3.1, qDTY 6.1, and qDTY 6.2, that show a high effect against the background of this variety. To create drought- and submergence-tolerant near-isogenic lines (NILs) of TDK1, Dixit et al. (2017) reported the pyramiding of these three QTLs with SUB1, which offers 2-3 weeks of resistance to total submergence. A tandem method was employed to create NILs with high yield in drought stress and non-stress situations as well as preferred grain quality. This technique combines marker-assisted backcross breeding with phenotypic selection. According to these findings, the most significant and reliable QTL impacting yield during drought circumstances is qDTY 3.1, followed by qDTY 6.1 and qDTY 6.2, respectively [206].

On the other hand, the limited predictive value of QT hinders the use of MAS in cereal breeding for improving quantitative aspects such as significant genotype–environment interaction, low expression of some genes, and recombination of markers and target genes [207]. Favorable individuals are chosen in genomic selection (GS-MAS) based on genomic estimated breeding values [208]. GS-MAS was recommended for accumulating favorable alleles with small additive effects and minor effects in later selection cycles [209]. Another method that has been applied with success is the backcrossing of alleles from the donor parent to the elite recurrent parent at one or more loci. Plant breeders have been utilizing marker-assisted backcrossing to choose the ideal characteristic, which involves alleles with high recurrent parent genome recovery. Sabitri, a Nepalese drought-tolerant rice variety, is one of MABC’s most successful examples [206]. MABC can be used to transfer characteristics from one variety to another in a variety of cereals. This method aids in the identification of QTL that are tightly linked to traits of interest [210].

## 5. Emerging Mapping and Technological Approaches in Cereal Breeding

Recent scientific breakthroughs have opened up a lot of new potential and advances in cereal breeding for desired traits. When compared to traditional cereal breeding procedures, novel molecular biology strategies have resulted in a significant rise in the development of better climate-resilient crop types. The new emerging plant breeding technologies and techniques for identification of QTL and candidate genes in cereals are presented in the following sections.

### 5.1. Genome-Wide Association Study (GWAS)

The GWAS is a powerful tool widely used in breeding programs because of its capability to quickly analyze complex features under a wide range of environments. It is frequently employed in conjunction with rapid advances in high-throughput sequencing methods for analyzing complex features in cereals. Low-temperature tolerance trait-associated loci in rice have been discovered using GWASs based on high-density SNP arrays [206,211]. The GWAS approach has helped researchers overcome the limits of bi-parental populations and improved genomic resolution, typically to the gene level. A recent study used a 15K wheat SNP assay for grain production and quality parameters in two heat-stressed locations, demonstrating persistent SNP markers on chromosomes 3B and 5A [212]. Hoang et al. (2019) used a panel of 180 rice landraces to conduct GWAS research to map various drought response and recovery traits. This research discovered 17 QTL related to a variety of drought responses and recovery traits during the vegetative stage, e.g., leaf relative water content, slope of relative water content, drought sensitivity score, recovery ability, and relative crop growth rate. As a result, utilizing huge populations and maps containing high marker density for a GWAS greatly enhanced QTL mapping resolution in cereals [209].

### 5.2. Clustered Regularly Interspaced Short Palindromic Repeats (CRISPR) 

Crop enhancement solutions using modern genome editing tools, such as clustered regularly interspaced short palindromic repeats-associated protein 9 (CRISPR/Cas9), are the way forward in cereal breeding. CRISPR/Cas9 developments have substantially accelerated agricultural breeding. Genome editing has become a highly valuable technique for crop improvement. Many QTL can influence grain production [213] and editing a QTL in a single independent or multiplex method can maximize yield [214]. As a result, genome editing technology may be implemented to manage the capacity to include some complicated features that are difficult to control using traditional breeding procedures. Haploid-inducer mediated genome editing (IMGE) and haploid induction edit (Hi-Edit) are two recent innovative rapid-breeding approaches that combine haploid induction with CRISPR/Cas9-mediated genome editing. These technologies introduce desired characteristics into dominant inbred lines within two generations, eliminating the time-consuming crossing and backcrossing methods [215].

Rice was one of the first plants to be used to demonstrate the viability of CRISPR-mediated targeted mutagenesis and gene replacement [216]. A study on CRISPR/Cas9-mediated QTL editing of five widely cultivated rice varieties, namely Nanjing 9108 (N9108), Wuyunjing 27 (W27), Yangjing 4227 (Y4227), Zhejing 22 (Z22), and Zhejing 88, uncovered that a grain size QTL, *GS3*, and grain number QTL, *Gn1a*, were altered. Long grain and increased grain yield phenotypes come from the loss of function of the organ size regulation (OSR) domain at the N terminus of GS3. *Gn1a* deficiency or loss of function leads to an increase in the number of reproductive organs of plants, which leads to increased grain output. Surprisingly, seven of the ten novel genotypes had lower grain yields than the wild type, demonstrating that the editing outcome was highly dependent on genetic background and emphasized the need for genetic diversity in varied environments [217]. CRISPR technology and its variants have been successfully implemented in cereals, ranging from studying gene function and protein localization to introducing desired traits, such as drought tolerance and increased grain size and number. For instance, plant annexins are essential for plant development and defense against environmental stressors. In *OsAnn3* CRISPR knockouts, the crucial role of the rice annexin gene (*OsAnn3*) during cold stress was investigated and the survival of T1 mutant lines was shown to be lower than that of wild-type plants [218]. The CRISPR/Cas9 technique was also successfully used to modify the genome of maize thermosensitive genic male-sterile 5 (*ZmTMS5*), which causes male sterility [219]. Protoplasts were used in a CRISPR/Cas9 genome editing method for two abiotic stress-related genes in wheat. The wheat assays for dehydration response element binding protein 2 (*TaDREB2*) and wheat ethylene responsive factor 3 (*TaERF3*) showed the success rate of this technique in modifying genes [220].

### 5.3. Meta-QTL Analysis for Stable QTL for Abiotic Stress Resistance

Meta-QTL (MQTL) analysis is a technique that combines QTL data from independent studies over different years, locations, and genetic backgrounds to find stable and consistent QTL [221]. When choosing MQTL, three requirements must be met: a small supporting interval, a large number of clusters of initial QTL, and a large influence of initial QTL on phenotypic variation [222]. Goffinet and Gerber (2000) created a meta-analysis method that works well with QTL data [223,224] which are useful for breeding programs. This approach determines the number of ‘real’ QTL most likely present in a QTL pool from many studies and offers consensus positions [225]. It is a valuable tool for comparing QTL from different studies and creating consensus map placements for QTL, allowing for the identification of QTL clusters for distinct characteristics and QTL hotspots for the same traits [226]. The MQTL analysis reveals the most stable QTL independent of the genetic background, phenotyping changes among locations and years, and marker density, which are the fundamental constraints of QTL mapping [227]. As a result, a single QTL may correlate to a large number of candidate genes. An MQTL analysis may now be performed using a variety of software tools. One of them is BioMercator, designed for scientists working on QTL mapping projects in any organism. MQTL analysis improves the accuracy of QTL position estimates by a factor of two compared to the original position of QTL in the same area [225]. MQTL are QTL found by meta-analysis from a stack of QTL with a 95% CI, which must be validated using a collection of germplasms or breeding lines. MQTL are beneficial for MAS because they have a small CI, are consistent, and greatly influence a trait [228].

Daware et al. (2017) discovered seven MQTL associated with grain weight from seven QTL studies on *indica* and aromatic rice accessions published between 2008 and 2015. In a study, MQTL4 and MQTL12 were significant MQTL for drought tolerance in wheat, with the potential to be employed in MAS breeding. These markers may be relevant for MAS since these MQTL are near the markers Xbarc5 and Xbarc154 [135]. The meta-analysis of the genomic regions that have been described in various studies aids in the determination of the most precise and confined genomic areas for use in the MAS introgression [229].

While meta-analysis has numerous advantages, it should be noted that the compiled studies differ significantly in their methodology, definition of independent and dependent variables, measurement techniques, data analysis procedures, and outcomes, leading to an incorrect conclusion. In many meta-analyses, the number of studies is small and such an approach is not feasible, suggesting that, when applied, these methods have important deficiencies [230]. When heterogeneity is high and its origins have not been adequately explored and addressed, combining many studies with methodological variations and varied impacts on results might be problematic [231].

Although QTL mapping is helpful for identifying complex trait architecture and candidate genes in defined QTL regions, techniques must be able to finely map the QTL with reliability and accuracy, especially in large complex genomes such as wheat and large populations with denser markers. This will ensure that the specified regions identified may be easily introgressed into recipient lines with higher efficiency and accuracy. Technology needs to advance for more time-, cost- and labor-effective plant breeding.

## 6. Conclusions

The primary goal of cereals is food production; hence, new strategies, such as enhanced stress tolerance in cereals, are necessary for boosting productivity to meet the global population’s anticipated food and energy requirements [232]. It is essential to increase agricultural plants’ resilience to stressors, as well as their yield and survival. Hence, it is crucial to understand and identify the factors that affect abiotic stress tolerance in cereals. It is important to understand the impact of abiotic stress on the cereal’s mechanisms and the various defense mechanisms at play in determining stable climate-resilient cereal production [233]. Plant breeders are primarily concerned with breeding plants that have desirable characteristics such as increased yield and tolerance to abiotic stresses. The application of genes and QTL in cereals that impart abiotic stress resistance helps increase yield under stress [234]. From what has been reviewed above, it would seem that molecular tools and techniques will be at the forefront of breeding programs in the future. Technological advancement in this area will be of great importance, where new cost- and time-efficient methods will be sought to meet agricultural product demand and reduce the negative effects of abiotic stressors and climate change. Hence, significant effort should be put into determining the essential traits for the growth rate, biomass output, and climate resilience of cereals [235]. The following are the ways forward in the development of climate-resilient cereal varieties.

- Improving technological advances: Accessibility of annotated genome sequences, cheaper and more efficient molecular markers, enhanced genomic selection prediction models, and breeding efficiency tactics can help put us in a unique position to meet the challenges ahead for cereal production. Many previously inaccessible traits can now be studied with MAS thanks to the availability of high-density markers and cheaper genotyping methods.
- Emerging molecular biology technologies: Integrating modern plant breeding technologies into current traditional breeding methods in cereals to provide sustainable yields in challenging climatic circumstances and the regarding the prevalence of abiotic stressors.
- Introduction of new genes: Enhancement of desirable features by mutation breeding, speed breeding, and quick generation advancements since all of these precision breeding methods can help improve certain traits during the breeding cycle

The above will enable the development of enhanced cereals with significantly better quality, boost cereal variety, increase yield, improve pest and disease resistance, boost nutritional quality, and make crops more climate resilient.

## 7. Literature Review Methodology

This review used the methodology of searching a huge number of studies and articles from search engines such as Google Scholar and PubMed. These articles and studies were filtered based on keywords mentioned in this manuscript related to rice, maize, and wheat for the main five abiotic stresses discussed in this manuscript and up to 553 articles were chosen from the search and readings. These articles were further filtered, and reduced to 232 suitable papers based on earlier important articles as well as the latest articles from 2016–2022. Among the landmark papers related to major QTL and plant breeding in these three cereals, those from Xu and Mackill (1996) [71], Bernier et al. (2007) [55], Vikram et al. (2011) [53], Uga et al. (2015) [25], Septiningsih et al. (2015) [67], and Würschum et al. (2017) [137] were used for critical analysis.

## Figures and Tables

**Table 1:** List of QTL linked to abiotic stress tolerance in rice.

Abiotic Stress	Population	Trait	Type of Markers	QTL/Gene/Marker	Chromosome/Marker	Reference
Drought	N22 × Swarna N22 × IR64 N22/MTU100	Grain yield	SSR	qDTY1.1	1	[53]
	IR74371-46-1-1 × Sabitri	Grain yield	SSR	qDTY12.1	12	[54]
	MRQ74 and MR219	Grain yield	SSR	qDTY12.1	12	[56]
	Vandana × Way Harem	Grain yield	SS	QTL2.1	12	[55]
	Sasanishika × Habataki	Flowering time	SSR	qDFT3	3	[20]
				qDFT8	8	
				qDFT10.1	10	
				qDFT11	11	
		Spikelet fertility	SSR	qSFht2	2	
				qSFht4.2	4	
		Pollen shedding	SSR	qPSLht1	1	
				qPSLht4.1	4	
				qPSLht5	5	
				qPSLht7	7	
				qPSLht10.2	10	
	13 parents	Grain yield	RFLP, SSR	qDTY1.1	1	[52]
			RFLP, SSR	qDTY2.2	2	
			RFLP, SSR	qDTY2.3	2	
			RFLP	qDTY3.1	3	
	IR64 × Kinandang Patong	Rice deep rooting	SSR	DRO1	7	[57]
	Cocodrie × N22	Grain number per panicle	SNP	qGN3.1	3	[51]
			SSR	qGN3.2	3	
			SSR	qGN5.1	5	
		Panicles per plant	SNP	qpn1.1	1	
		Grain yield	SNP	qGY1.1	1	
			SSR	qGY7.1	7	
			SSR	qGY8.1	8	
			SNP	qGY11.1	11	
Cold stress	Kuchum × Hitomebore	Seed fertility	SSR	qCT-4	4	[62]
	Ukei 840 × Hitomebore	Seed fertility	-	qLTB3	3	[63]
	BR1 × Hbj.BVI	Cold tolerance seedling stage	SSR	qCTSL-8-1	8	[29]
			SSR	qCTSL-12-1	12	
			SSR	qCTSS-8-1	8	
			SSR	qCTCC-12-1	12	
	Huanghuazhan	Cold tolerance booting stage	SNP	qCT-3-2	3	[64]
	Dongnong422 × Kongyu131	Percent seed set	SSR	qPSST6	6	[90]
Flooding stress	ID72 × Madabaru	Submergence tolerance	SSR	qSub1.1	1	[70]
			SSR	qSub2.1	2	
			SSR	qSub9.1	9	
			SSR	qSub12.1	12	
	IR40931-26 × PI543851FR13A	Dry weight	-	Sub1A	9	[71]
Salinity stress	Pokkali × IR29	Na/K^+^ absorption rate	RFLP	*Saltol*	1	[72]
	IR36 × Weiguo	Relative shoot length	SNP	qRSL7	7	[76]
	Wujiaozhan × Nipponbare	Germination rate	-	qGR6.2	6	[77]
	IR26 × Jiucaiqing	Seed germination	SNP	qSE3	3	[78]
Heavy metal stress	‘Suwon490′ × ‘SNU-SG1′	Shoot for Cd accumulation	-	scc10	10	[82]
		Grain for Cd accumulation	-	gcc3	3	
			-	gcc9	9	
			-	gcc11	11	
	Xiang 743 × Katy	Cd concentration	SSR	qCd-2	2	[85]
			SSR	qCd-7	7	
	Tainan1 (TN1) × Chunjiang06	Cd concentration	-	CAL1	1	[83]
	Nipponbare × Anjana Dhan	Cd accumulation	SSR	RM8006	7	[91]
	Dhusura × Sebati	Fe toxicity tolerance	SSR	qFeTox4.3	4	[92]
			SSR	qFeTox6.1	6	
			SSR	qFeTox10.1	10	
	*O. glaberrima* × *O. sativa*	Fe concentration in leaf blade	SSR	RM5-RM246	1	[87]
	413 inbred accessions	Grain As concentration	SNP	qGAS1	1	[93]
	WTR1 × Hao-an-nong	As content in shoot	SNP	qAsS2	2	[88]
		As content in shoot	SNP	qAsS5.1	5	
			SNP	qAsS5.2	5	
			SNP	qAsS6	6	
			SNP	qAsS9.1	9	
			SNP	qAsS9.2	9	
		As content in root	SNP	qAsR8.1	8	
			SNP	qAsR8.2	8	
		Chlorophyll content	SNP	qRChlo1	1	
	Yuefu × IRAT109	Root length	SSR	qRRL-1	1	[89]
			SSR	qRRL-2	2	
			SSR	qRRL-5	5	

**Table 2:** List of QTL linked to abiotic stress tolerance in maize.

Abiotic Stress	Population	Trait	Type of Markers	QTL/Gene/ Marker	Chromosome/ Marker	Reference
Drought	Langhuang × TSI41	Ear height to plant height ratio	RFLP	qEHPH-Ch.3-1	3	[102]
		Grain weight	RFLP	qGW-Ch.1-2	1	
			RFLP	qGW-Ch.1-1	1	
			RFLP	qGW-J1-1	1	
			RFLP	qGW-Ch.4-1	4	
			RFLP	qGW-Ch.8-1	8	
			RFLP	qGW-J8-1	8	
		Kernel ratio	RFLP	qKR-Ch.1-2	1	
			RFLP	qKR-J1-1	1	
	H082183 × Lv28	Ear weight	-	qEW1s	1	[103]
		Hundred-kernel weight	-	qHKW7s	7	
	DH1M × T877	Crown root angle	SNP	CRA1	1	[104]
			SNP	CRL1	1	
	DTPWC9F104 × LPSC7F64	Senescence (6 weeks after flowering)	SNP	-	2	[105]
	CML444 × MALAWI, CML440 × CML504, CML444 × CML441	Stay green	SNP	-	3	[19]
Cold stress	Tohoku-PL3 × Akihikari	Spikelet fertility	RFLP	qCTR5	5	[110]
			RFLP	qCTR12	12	
	B73 × P39 B73 × IL14h	Vigor	SNP	-	4	[108]
		Ear height	SNP	-	8	
	B73 × Mo17 (IBM)	Germination rate	RFLP	qLTGR5-1	5	[109]
		Root length	RFLP	qLTPRL9-1	9	
	B73 × Mo17 (IBM)	Plumule length	-	qLTPL1-1	1	[112]
		Seedling length	-	qLTSL1-1	1	
	80 inbred lines W72 × W10	Peroxidase activity at seedling stage	SNP	qPOD3	3	[111]
Submergence stress	HZ32 × K12	Plant height	SSR	ph1-1	1	[114]
			SSR	ph1-3	1	
		Shoot dry weight	SSR	sdw9-1	9	
		Total dry weight	SSR	tdw9-1	9	
			SSR	tdw9-2	9	
			SSR	tdw9-3	9	
		Root dry weight	SSR	rdw9-2	9	
	Mo18W × B73	Submergence tolerance trait	-	Subtol6	6	[115]
Salinity stress	PH6WC × PH4CV	Plant height	SNP	qSPH1	1	[119]
	Xianyu335 (PH6WC × PH4CV)	Root length	SNP	qRLS1	1	[120]
		Shoot length	SNP	qSLS1-2	1	
		Full length	SNP	qFLS1-2	1	
		Root fresh weight	SNP	qRFS1	1	
		Full fresh weight	SNP	qFFS1	1	
		Root length	SNP	qRLR1	1	
	Zheng58 × Chang7-2	Leaf Na^+^ and K^+^ contents	-	*ZmNC1*	3	[121]
Heavy metal stress	Zong3/87-1 × Yuyu22	Kernel As concentration	RFLP	XAsK1a	1	[122]
	IBMSyn10 DH	Leaf Cd accumulation	SNP	qLCd2	2	[123]
	B73 × Mo17	Root fresh weight (Pb ad Cd tolerance coefficient)	-	qRFWLCTC2-1	1	[124]
		Shoot height (Pb and Cd tolerance coefficient)	-	qSHLLCTC2-2	2	

**Table 3:** List of QTL linked to abiotic stress tolerance in wheat.

Abiotic Stress	Population	Trait	Type of Markers	QTL/Gene/ Marker	Chromosome/Marker	Reference
Drought	Cranbrook × Halberd	Osmotic stress Spike	SNP	IWB72377	2A	[21]
		Stress tolerance trait	SNP	*VRN-A1*	5A	
	Colosseo × Lloyd Meridiano × Claudio	Seminal root angle	SNP	QRga.ubo-2B	2B	[133]
				QRga.ubo-4B	4B	
				QRga.ubo-6A	6A	
	SYN-D (Croc 1/*Aegilops squarrosa* (224)//Opata) × Weebill 1	Thousand-grain weight, grain number	SNP	QTGW-2A.1	2A	[24]
		Yield	SNP	QYLD-3D.1	3D	
			SNP	QYLD-6D.1	6D	
			SNP	QYLD-6D.2	6D	
			SNP	QYLD-7B.1	7B	
	Excalibur × Kukri	Yield	-	QYld.aww-1B.2	1B	[129]
	Chinese Spring × SQ1 (Highbury × TW269/9/3/4)	Yield	SSR	Qyld.csdh.7AL	7A	[130]
	DBA Aurora × Fastoz8	Seminal root angle	DArT	qSRA-6A	6A	[132]
	Reeder × Albany	Thousand-kernel weight	SNP	QTW.ndsu.7B	7B	[134]
		Yield	SNP	QYL.ndsu.2B	2B	
			SNP	QYL.ndsu.7B	7B	
Cold stress	*Triticum spelta* × Cheyenne	Frost resistance	RFLP	*Fr1*	5A	[136]
	*Triticum spelta 5A* × Cheyenne 5A	Frost resistance	-	*FR2*	5D	[138]
	-	Frost resistance	RFLP	FR-2	5A	[137]
	Norstar × Winter Manitau	Low-temperature tolerance	SNP	QLT50.usw-5A.1nm	5A	[139]
				QLT50.usw-5A.2nm	5A	
	Capelle Desprez × Norstar	Low-temperature tolerance	SNP	QLT50.usw-5A.1nc	5A	
	Norstar × Winter Manitau	Low-temperature tolerance	SNP	QLT50.usw-5A.1	5A	
Submergence stress	W7984 × Opata85	Germination rate index	SSR	Xfbb264	7A	[144]
	USG3209 × Jaypee	Chlorophyll content	-	QSpad3.ua-1D.5	1D	[145]
Salinity stress	Kharcia65 × HD2009	Plant height	SSR	QSph.iiwbr-6A	6A	[147]
		Date of flowering	SSR	QSdth.iiwbr-2D	2D	
	Line 149 × Tamaroi	Leaf blade low Na+ concentration	AFLP, RFLP	NAX1	2A	[148]
	WTSD91 × WN-64	Na+ exclusion	SNP	qSNAX.2A.1	2A	[149]
			SNP	qSNAX.2A.2	2A	
			SNP	qSNAX.7A.3	7A	
			SNP	qRNAX.7A.3	7A	
Heavy metal stress	Grenora × Haurani	Grain Cd content	SNP	IWA1775	5B	[152]
	D041735 × Divide	Cd absorption	SNP	QCdu.ndsu-5B	5B	[153]
	UI Platinum × LCS Star	Cd content in grain	SNP	QCd.uia2-5B	5B	[154]
			SNP	QCd.uia2-7B	7B	
			SNP	QCd.uia2-7D	7D	
	Chinese spring × ‘Synthetic 6x’	Al tolerance	SSR	Xgdm125-Xgwm976	4D	[155]
			SSR	Qalt cs.ipk-3B	3B	

## Data Availability

Not Applicable.
